# 

**Published:** 1889-06

**Authors:** 


JUNE, 1889.

*Vol. VII.* *No.* **24.**

THE

### BRISTOL MEDICO-CHIRURGICAL JOURNAL

**A Quarterly Journal of the Medical Sciences for the West of England and South Wales**

*PUBLISHED UNDER THE AUSPICES OF*

*THE BRISTOL MEDICO-CHIRURGICAL SOCIETY.*

LIBRARY OF CONGRESS
574777
DEC 1 1897
SMITHSONIAN DEPOSIT

EDITOR:

J. GREIG SMITH, M.A., F.R.S.E.

ASSISTANT-EDITOR:

L. M. GRIFFITHS.

"Scire est nescire, nisi id me

Scire alius sciret."

BRISTOL: J. W. ARROWSMITH;

LONDON: J. & A. CHURCHILL, 11 NEW BURLINGTON STREET.

*Price One Shilling and Sixpence.*

